# Persulfidation is the mechanism underlying sulfide-signaling of autophagy

**DOI:** 10.1080/15548627.2021.1936357

**Published:** 2021-06-07

**Authors:** Cecilia Gotor, Angeles Aroca, Luis C. Romero

**Affiliations:** Instituto de Bioquímica Vegetal y Fotosíntesis, CSIC-Universidad de Sevilla, Sevilla, Spain

**Keywords:** Arabidopsis, GAPDH, hydrogen sulfide, persulfidated cysteine, posttranslational modification, stress

## Abstract

In this commentary, we highlight the findings described in a recent paper regarding the mechanism of H_2_S regulation of macroautophagy/autophagy in mammalian cells and discuss the similarities/divergencies with plant cells. The main outcome is that the posttranslational modification of thiol groups of cysteine residues to form persulfides is a conserved molecular mechanism.

Hydrogen sulfide is now a well-established relevant signaling molecule in both animal and plant physiology, and with the same relevance as nitric oxide, carbon monoxide and hydrogen peroxide [1–6]. Hydrogen sulfide, (hereafter referred to as H_2_S) includes the neutral H_2_S and the HS^−^and S^2-^ anionic forms present in aqueous neutral pH solutions [7]. H_2_S is precisely produced and metabolized by cells and regulates extensive vital processes in both animal and plant systems [2,8]. Regardless of the increasing studies on H_2_S biological functions, the molecular mechanism of its action in any particular physiological process is yet scantly known, and undoubtedly it must be founded on the chemical reactivity of H_2_S. The mechanism that is predominantly demonstrated in recent years is the modification of the thiol groups of cysteines mediated by H_2_S-forming persulfide groups (-SSH), thus resulting in a reactivity change of the cysteines that can originate alterations in protein structure, enzymatic activity, or subcellular localizations [9,10]. This posttranslational modification named persulfidation (also known as S-sulfhydration), has been demonstrated as the regulating mechanism by H_2_S of essential processes for animals and plants (as examples of most recent studies see [11–13]).

In the recent study published by Iqbal et al. [14], persulfidation was demonstrated as the underlying mechanism of the regulation of autophagy by H_2_S in mammalian cells, as likewise was previously highlighted in the plant *Arabidopsis thaliana* [13]. Thereby, this posttranslational modification may be a conserved mechanism of H_2_S signaling to regulate autophagy in eukaryotic cells. Nevertheless, the H_2_S regulation of autophagy appears to proceed in opposite directions. Whereas the Iqbal et al. investigation [14] demonstrated the H_2_S-mediated activation of autophagy, previous studies in Arabidopsis showed the role of sulfide as a repressor of autophagy [15–17]. However, the final outcome of the H_2_S signaling seems to be the same in both living systems, which is a protection effect for survival.

In animal systems, the link between H_2_S and autophagy has been described in different pathologies and H_2_S acts as an activator or a repressor depending on the pathology, although in all cases described, the final role of H_2_S is protecting cells [18]. Similarly, Iqbal et al. [14] brightly demonstrated that H_2_S-mediated induction of autophagy is essential for the trafficking of *Mycobacterium tuberculosis* cells into lysosomes to restrict the intracellular mycobacteria growth, and consequently results in better health outcomes. In plants, particularly in Arabidopsis, the interplay between H_2_S and autophagy has been studied under stress conditions. H_2_S functions as a signaling molecule negatively regulating autophagy induced upon nutrition limitation, either in leaves under dark-induced carbon starvation [16] or in roots under nitrogen deprivation [15]. In both cases, the negative regulation of autophagy by sulfide also results in a reduction in the typical phenotypic characteristics under stress. In a study aimed to decipher the molecular mechanism involved in the H_2_S signaling of plant autophagy, it was also shown to have a role in repressing the autophagic flux induced by abscisic acid (ABA) treatment [13]. The phytohormone ABA increases its concentration when plants are subjected to abiotic stress conditions in order to activate an ABA-signaling pathway and induce downstream effectors that regulate diverse physiological processes [19]. Thus, when plants are exposed to adverse environmental conditions, a battery of responses are activated to cope with stress and promote survival, including the induction of autophagy [20]. In this particular context, the presence of H_2_S during stress prevents the activation of autophagy to the levels observed in favorable growth conditions, and therefore plays a role in improving plant performance. In a very recent study, the role of H_2_S in the regulation of autophagy has also been demonstrated in another stress condition in plants. Autophagy is activated in response to endoplasmic reticulum (ER) stress, which can be triggered in plants by different adverse environmental conditions. Thus, H_2_S was revealed as a negative regulator of autophagy induced by ER stress [21], confirming that the final outcome of H_2_S is allowing plant survival.

The molecular mechanism by which H_2_S regulates autophagy, hence, has been established to be the persulfidation of specific cysteines on target proteins, both in mammalian [14] and plant systems [13,21]. In the mammalian study, the persulfidation of the active site cysteine of the glycolytic GAPDH (glyceraldehyde-3-phosphate dehydrogenase) induces the translocation of the protein to the nucleus, leading to the deacetylation of the autophagic core protein LC3B, resulting in autophagosome formation and the progression of autophagy. In Arabidopsis, the target protein is the Cys protease ATG4 which cleaves the C-terminal extension of ATG8 (LC3 and GABARAP ortholog) that is essential for the formation of autophagosomes. Under basal conditions, persulfidation of the catalytic cysteine residue of ATG4 inhibits its proteolytic activity and consequently autophagy initiation. An increase in the intracellular level of ABA transiently decreases the level of ATG4 persulfidation and consequently favors the processing of ATG8 to allow lipidation and autophagy progression. Under an ER-stress situation, persulfidation of the core autophagic protein ATG18a at cysteine residue 103 activates its binding capacity to phospholipids, consequently increasing its binding to membranes and delaying its release, and therefore avoiding autophagosome maturation and progression of autophagy [21]. Collectively, persulfidation of GAPDH on the one hand, and the lack of persulfidation of ATG4 and of ATG18a on the other hand, activate autophagy. The rationale behind this apparent contradiction, either stimulating or repressive effects of persulfidation is unknown, but we can speculate that probably the effect relies on the specific protein target which is persulfidated. Particularly, persulfide residues are more nucleophilic and acidic and, therefore, more reactive than the original thiols, and most likely the location of the residue in the protein structure (specific domain/catalytic site) must determine the final outcome.

Another intriguing aspect worthy of discussion is related to the mechanism that connects the required H_2_S production to generate protein persulfidation under different stress conditions in both animal and plant cells. At present this angle has been unexplored; nevertheless, there are some clues indicating that upon an adverse condition the induction of H_2_S-generating enzymes must occur. Several studies on Arabidopsis guard cells have demonstrated that ABA triggers the induction of DES1 (L-cysteine desulfhydrase 1), a cytosolic enzyme involved in the degradation of cysteine and the concomitant generation of H_2_S. As a consequence, the guard cell H_2_S level is raised, and, by a persulfidation-based mechanism, stomatal closure occurs as a plant adaptation to adverse conditions such as drought stress [12,22–24]. In animal cells, persulfidation is controlled by H_2_S, mainly produced through the enzymes involved in the transulfuration pathway, CBS (cystathionine beta-synthase) and CTH/CSE (cystathionine gamma-lyase), together with MPST/3-MST (mercaptopyruvate sulfurtransferase). Extensive reports have shown that the levels of these enzymes are closely related to different diseases. Thus, the loss/decrease of these enzymes in neurodegenerative diseases and during aging is observed, resulting in a loss of protein persulfidation [5,11].

Persulfidation of the GAPDH protein is another intriguing aspect that arises from the Iqbal et al. study [14]. Different experimental approaches such as, expression of GFP-tagged GAPDH and subcellular fractionation show the nuclear localization of GAPDH upon H_2_S exposure. Interestingly, by using the same experimental approaches, it was also concluded that H_2_S enhances the nuclear localization of the cytosolic GAPDH in Arabidopsis [10], although the connection of the GAPDH translocation and autophagy was not investigated in this study. In addition, while the persulfidated cysteine in mammalian GAPDH is the active site Cys150 [14], the modified cysteine in plant GAPDH involving nuclear translocation is a second cysteine present in the sequence, which is in close proximity to the active site [10].

In conclusion, the findings present in the Iqbal et al. manuscript [14] on the H_2_S signaling of autophagy in an animal system, together with previous findings acquired in plant studies, highlight persulfidation as a molecular mechanism underlying autophagy regulation, being a conserved mechanism in both eukaryotes. Conversely, what appears to be divergent are the specific protein targets that may be susceptible to be persulfidated, at least with the present knowledge. However, we are confident that future research will increase the identification of additional targets of sulfide and perhaps also reveal the extended conservation of this process in different eukaryotic systems.
